# An Inducible Chaperone Adapts Proteasome Assembly to Stress

**DOI:** 10.1016/j.molcel.2014.06.017

**Published:** 2014-08-21

**Authors:** Ariane Hanssum, Zhen Zhong, Adrien Rousseau, Agnieszka Krzyzosiak, Anna Sigurdardottir, Anne Bertolotti

**Affiliations:** 1 MRC Laboratory of Molecular Biology, Francis Crick Avenue, Cambridge CB2 0QH, UK

## Abstract

The proteasome is essential for the selective degradation of most cellular proteins. To survive overwhelming demands on the proteasome arising during environmental stresses, cells increase proteasome abundance. Proteasome assembly is known to be complex. How stressed cells overcome this vital challenge is unknown. In an unbiased suppressor screen aimed at rescuing the defects of a yeast Rpt6 thermosensitive proteasome mutant, we identified a protein, hereafter named Adc17, as it functions as an ATPase dedicated chaperone. Adc17 interacts with the amino terminus of Rpt6 to assist formation of the Rpt6-Rpt3 ATPase pair, an early step in proteasome assembly. Adc17 is important for cell fitness, and its absence aggravates proteasome defects. The abundance of Adc17 increases upon proteasome stresses, and its function is crucial to maintain homeostatic proteasome levels. Thus, cells have mechanisms to adjust proteasome assembly when demands increase, and Adc17 is a critical effector of this process.

## Highlights

• The maintenance of adequate levels of proteasome is vital
• Cells increase proteasome abundance when the needs increase
• Adc17 is an inducible chaperone that adapts proteasome assembly to increased needs
• Adc17 assists the pairing of Rpt6 and Rpt3, an early step in proteasome assembly

## Graphical Abstract

**Figure:**
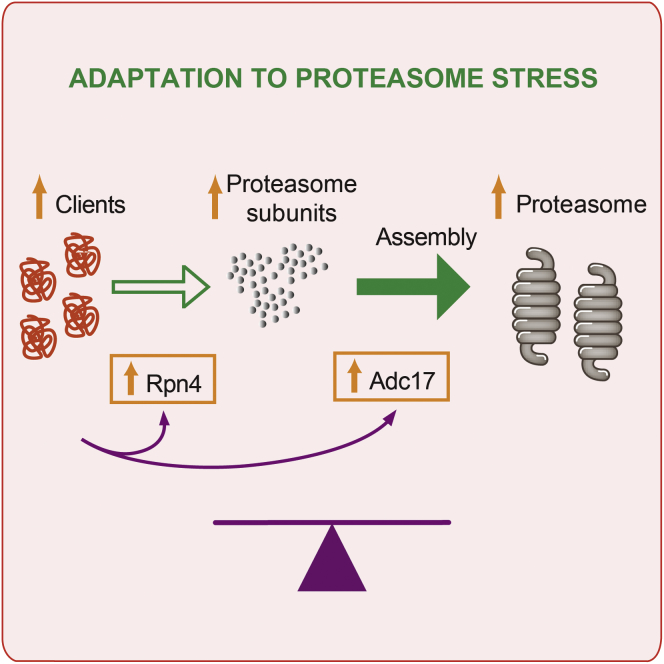

## Teaser

Cells increase proteasome abundance in order to survive during environmental stress. Hanssum et al. reveal a mechanism for controlling proteasome abundance, showing that Adc17 is an inducible chaperone needed for proteasome assembly during stress.

## Introduction

Cells normally strive to ensure that proteins get correctly folded and that damaged, mutant, or misfolded proteins are eliminated. To maintain protein homeostasis under adverse conditions, cells have evolved powerful and sophisticated protein quality control systems that normally operate very efficiently (Kim et al., 2013). However, increasing evidence suggests that protein quality control gradually fails with age, which has devastating consequences for cells and organisms (Kim et al., 2013, Morimoto, 2011). Indeed, accumulation of misfolded, aggregation-prone proteins is the hallmark of a broad range of human diseases (Cuanalo-Contreras et al., 2013, Kim et al., 2013, Morimoto, 2011, Soto, 2003). Identifying strategies to improve the cells’ ability to handle misfolded proteins could not only reveal novel aspects of the cellular defense systems against misfolded proteins but also uncover novel strategies to correct the diverse disorders that arise when protein quality control fails.

The proteasome is a key component of protein quality control systems that degrades a large number of cellular proteins and thereby controls virtually all cellular processes (Hershko and Ciechanover, 1998, Goldberg, 2007, Finley, 2009, Tanaka et al., 2012). The proteasome is evolutionarily conserved, and inhibition of the proteasome is lethal in all species (Kisselev and Goldberg, 2001, Navon and Ciechanover, 2009). When the proteasome is inhibited, cells accumulate undegraded proteins (Kisselev and Goldberg, 2001, Navon and Ciechanover, 2009). In addition, proteasome inhibition causes a lethal amino acid imbalance, since the undegraded proteins immobilize a pool of amino acids that would otherwise be recycled (Suraweera et al., 2012). Failure of the ubiquitin-proteasome system has been associated with a broad range of pathological conditions, such as the devastating neurodegenerative diseases characterized by the age-dependent deposition of aggregation-prone proteins (Sherman and Goldberg, 2001, Schwartz and Ciechanover, 2009).

Since the maintenance of adequate levels of proteasomes is vital for cells and organisms, the abundance of functional proteasome must be tightly regulated to enable cells to adapt and survive changes in their environment, in particular those that overwhelm the proteasome. Indeed, it has been previously established that cells increase expression of proteasome subunits in a concerted manner when the demand for proteasomes increases, for example during protein misfolding stress (Hanna and Finley, 2007). In yeast, this is controlled by Rpn4, a transcription factor that regulates expression levels of proteasome subunits via a homeostatic negative feedback loop (Xie and Varshavsky, 2001). Rpn4 is an unstable protein that is normally rapidly degraded but accumulates when the proteasome is overwhelmed (Hanna and Finley, 2007). Increasing the levels of proteasome subunits, while necessary, is not sufficient to increase proteasome levels. Indeed, the levels of functional proteasome depend not only on the expression of its subunits but also on their precise assembly.

Recent studies have shed light on the pathways for the ordered assembly of the proteasome (Murata et al., 2009, Tomko and Hochstrasser, 2013). Proteasome assembly is an extremely complex and challenging process in the crowded cellular environment that requires the precise arrangements of the 33 subunits (Murata et al., 2009, Tomko and Hochstrasser, 2013). An understanding of the assembly of the regulatory particle (RP) of the proteasome began recently with the discovery of assembly intermediates and chaperone assembly factors (Funakoshi et al., 2009, Le Tallec et al., 2009, Roelofs et al., 2009, Saeki et al., 2009). The six ATPases of the RP, Rpt1–Rpt6, assemble in a unique order upon formation of a trimer of specific pairs of ATPases (Förster et al., 2009, Tomko et al., 2010). The yeast RP assembly chaperones Nas2, Nas6, Rpn14, and Hsm3 regulate base assembly by binding to the carboxyl termini of Rpts, thereby preventing binding of immature complexes to the core particle (CP) (Park et al., 2013). While ATPase modules composed of Rpt pairs bound to their carboxy-terminal chaperones have been isolated, it is unknown how Rpts find their dedicated partners.

Proteasome assembly is challenging under normal conditions. How cells manage to assemble proteasomes under stress conditions associated with an accumulation of undesirable proteins is unknown. This represents a problem of vital importance, since cell and organismal survival depends on adequate levels of functional proteasomes.

Through an unbiased screen in yeast designed to identify suppressors of proteasome defects, we discovered a previously uncharacterized stress-induced protein, Adc17, which facilitates early steps in RP assembly. Adc17 is crucial to adapt proteasome assembly to increased demands. The discovery of this stress-inducible chaperone reveals that proteasome assembly is an adaptive process.

## Results

### Identification of *ADC17* as a Suppressor of the *cim3-1* Defects

**Figure 1:**
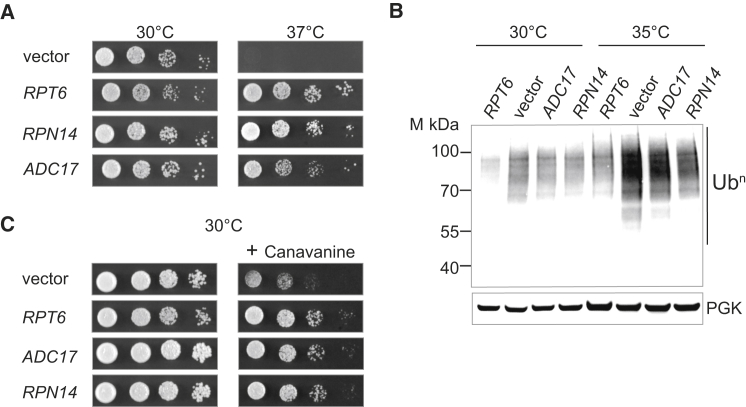
Adc17 Suppresses the Defect of the *cim3-1* Proteasome Mutant (A) Expression of *ADC17*, *RPN14*, or wild-type *RPT6* suppresses the temperature-sensitive growth defect of the *cim3-1* mutant. Cells were spotted in 6-fold dilution and grown at the permissive (30°C) or restrictive (37°C) temperature. (B) Polyubiquitin (Ub^n^) or phosphoglycerate kinase (PGK) immunoblots from lysates of *cim3-1* cells expressing *RPT6*, *ADC17*, *RPN14*, or vector alone (p416 GPD) grown at the indicated temperatures. (C) Yeast cells (*cim3-1*) transformed with the indicated plasmids or empty vector (p416 GPD) were serially diluted and spotted on selective medium in the presence of canavanine (1 μg/ml) where indicated.

Aiming at understanding how cells adapt to proteasome deficiencies, we performed an unbiased genome-wide multicopy suppressor screen in the yeast *rpt6* thermosensitive proteasome mutant *cim3-1* (Ghislain et al., 1993, Suraweera et al., 2012). In addition to wild-type *RPT6*, we isolated two potent suppressors of the *cim3-1* growth defects (Figure 1A). One encoded the previously reported suppressor, *RPN14* (Funakoshi et al., 2009), an assembly factor that binds to the carboxyl terminus of Rpt6. The second potent suppressor of the *cim3-1* defects was *TMA17*, encoding a protein of unknown function, previously found in association with ribosomes (Fleischer et al., 2006). We renamed the protein Adc17 for ATPase dedicated chaperone of 17 kDa, following its functional characterization, as will be described below.

High-copy *ADC17* rescued growth of *cim3-1* at nonpermissive temperature (Figure 1A), but not of the *rpt1 ts* mutant *cim5-1* (Ghislain et al., 1993) or the CP *ts* mutant *pre1-1* (Heinemeyer et al., 1991) (Figure S1). This suggests a genetic interaction between *RPT6* and *ADC17*. Increased accumulation of polyubiquitin conjugates and sensitivity to canavanine, an arginine analog, are hallmarks of proteasome mutants. High-copy *ADC17* or *RPN14* markedly reduced accumulation of polyubiquitin conjugates in the *cim3-1* cells at 30°C and 35°C (Figure 1B) and abrogated the canavanine sensitivity of the *cim3-1* (Figure 1C). These results suggest that the function of Adc17, a previously uncharacterized protein, is related to the proteasome.

### Adc17 Is Important for Cell Fitness

**Figure 2:**
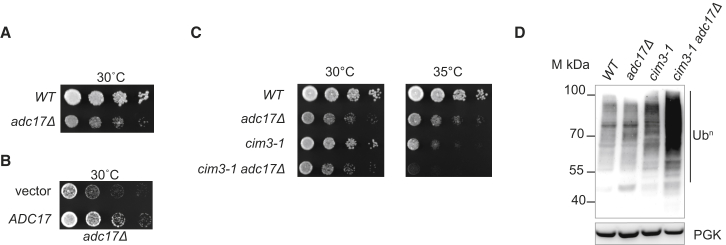
Deletion of *ADC17* Compromises Cell Growth and Aggravates the *cim3-1* Defects (A–C) Cells of the indicated genotype were spotted in 6-fold dilution and grown at the indicated temperature. (D) Polyubiquitin (Ub^n^) or phosphoglycerate kinase (PGK) immunoblots from lysates of cells of indicated geneotypes grown at 30°C.

To further investigate whether the function of Adc17 was related to the proteasome, we next deleted *ADC17.* The deletion of *ADC17* decreased cell fitness (Figure 2A), and this defect was specific as it was rescued upon overexpression of *ADC17* (Figure 2B). In the *cim3-1* cells, deletion of *ADC17* caused severe growth defects (Figure 2C), and this was associated with a proteasome defect, as manifested by an overload of polyubiquitinated conjugates in the *cim3-1 adc17Δ* cells (Figure 2D). These results reveal that Adc17 is important for cell fitness and that its function is related to the proteasome.

### Adc17 Is Important for Proteasome Integrity

**Figure 3:**
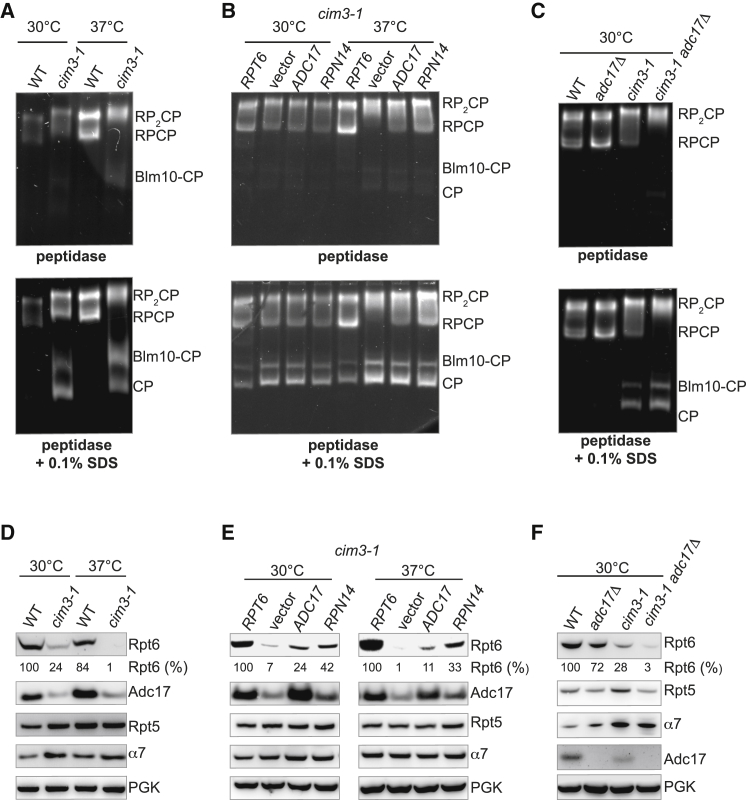
Adc17 Is Important for Proteasome Integrity (A) Native-PAGE (4.2%) of yeast extracts of wild-type (WT) or *cim3-1* cultures, grown at 30°C or 37°C, revealed with the fluorogenic substrate Suc-LLVY-AMC. RP_2_CP, doubly capped proteasome; RPCP, singly capped proteasome. The alternative proteasome complex composed of the activator Bml10 bound to CP is indicated. Addition of 0.1% SDS activated the otherwise latent free CP. (B) Same as in (A) with *cim3-1* cells transformed with the indicated plasmids or empty vector. *RPT6*-complemented cells serve as a reference. (C) Native-PAGE of yeast extracts of cells of the indicated genotype, grown at 30°C. (D–F) Quantification of Rpt6 levels, normalized to control (either wild-type or Rpt6 complemented cells), are shown below Rpt6 blots. (D) Immunoblots of total extracts of wild-type or *cim3-1* cells grown at 30°C or 37°C. (E) Same as in (D) with *cim3-1* transformed with the indicated plasmids or empty vector. (F) Immunoblots of total extracts of cells of the indicated genotype grown at 30°C. Each panel shows representative results of an experiment repeated independently three times.

To characterize the function of Adc17, we next analyzed proteasome integrity by in-gel peptidase assays on whole-cell lysates. Active 26S proteasomes exist as a mixture of CP bound to one or two RP, but the specific functional properties of RPCP and RP_2_CP are currently unknown (Finley, 2009). As expected, both RPCP and RP_2_CP were detected in wild-type cells, and these two complexes appeared equally abundant (Figure 3A). The *cim3-1* cells at 30°C also contained similar amounts of RPCP and RP_2_CP (Figure 3A). While equal amounts of total proteins were loaded in each condition (see Experimental Procedures for details), the abundance of 26S proteasome (RPCP and RP_2_CP) appeared consistently higher than in the wild-type cells (Figure 3A). This attests of a proteasome defect in the *cim3-1* cells, as already noticed with the accumulation of polyubiquitinated conjugates in this mutant (Suraweera et al., 2012 and Figure 1B). Cells attempt to compensate for proteasome defects by increasing proteasome levels (Xie and Varshavsky, 2001). At 37°C, the proteasome profiles of the *cim3-1* cells were markedly abnormal, with a nearly complete loss of RPCP complexes (Figure 3A). In the *cim3-1* cells, the 26S proteasome complexes had reduced mobility relative to their wild-type counterparts, further highlighting proteasome abnormalities in the mutant cells. Together, the results establish that the proteasome is abnormal in *cim3-1*. Free CP, revealed with low concentration of SDS (Kisselev and Goldberg, 2005), hardly detectable in the wild-type strain at either 30°C or 37°C, increased in *cim3-1* cells (Figure 3A), confirming that the proteasome is defective in the mutant cells. The suppressors *ADC17* or *RPN14* noticeably increased the levels of RPCP in the *cim3-1* at 37°C, although free CP remained (Figure 3B). These results reveal that the proteasome is defective in *cim3-1* cells and overexpression of *ADC17* or *RPN14* partly rescued 26S proteasome defects. Deletion of *ADC17* caused a modest but reproducible increase in the levels of both RPCP and RP_2_CP (Figure 3C), similar to what was observed for the *cim3-1* mutant at 30°C (Figure 3A). This suggests that deletion of *ADC17* causes proteasome defects to which cells adapt by increasing proteasome levels. Furthermore, the deletion of *ADC17* impaired the proteasome of the *cim3-1* cells, as *cim3-1 adc17Δ* cells completely lacked RPCP already at 30°C (Figure 3C). These findings explain the proteolytic defects observed in *cim3-1*, their rescue upon Rpn14 and Adc17 overexpression, and the aggravated proteasome defects of the *cim3-1 adc17Δ* (Figures 1 and 2). Together, these results establish that Adc17 is important for proteasome integrity.

To understand the molecular basis of the observed proteasome defects, we next examined the levels of different proteasome subunits in different cells. We found that the levels of Rpt6 were dramatically decreased in the *cim3-1* cells (*rpt6* thermosensitive mutant) at 30°C and even more at 37°C (Figure 3D). In contrast to the protein levels, the levels of mRNA encoding Rpt6 were significantly higher in *cim3-1* (Figure S2). This implies that Rpt6 is destabilized by the ts mutation in *cim3-1*, and cells attempt to compensate for this defect by increasing Rpt6 mRNA. In contrast, the protein levels of Rpt5 or the CP subunit α7 rather increased in *cim3-1* (Figure 3D). The expression of the suppressors *RPN14* and *ADC17* specifically increased the abundance of Rpt6 in *cim3-1* cells at both at 30°C and at 37°C (Figure 3E). Since Rpt6 is essential (Rubin et al., 1998), the absence of Rpt6 at 37°C in *cim3-1* explains the growth defects, and restoring viable levels of Rpt6 upon Adc17 or Rpn14 overexpression in *cim3-1* provides the basis for the rescued growth (Figure 1A) and rescued proteasome function (Figure 3B). Interestingly, the levels of Adc17, revealed with a specific Adc17 antibody (Figure S3), were also reduced in *cim3-1* (Figure 3D), and overexpression of Rpt6 rescued this defect without noticeably altering the levels of Rpt5 or α7 (Figure 3E). As previously noticed (Figure 3D), the levels of Rpt6 were reduced in *cim3-1* (Figure 3F). Deletion of *ADC17* in the *cim3-1* cells further reduced the levels of Rpt6 (Figure 3F). Thus, Adc17 is important to maintain adequate levels of Rpt6 required for proteasome integrity (Figures 3A–3C) and to sustain cell viability (Figure 2).

### Adc17 Is Not Part of the Proteasome but Is Engaged in a Complex with Rpt6

**Figure 4:**
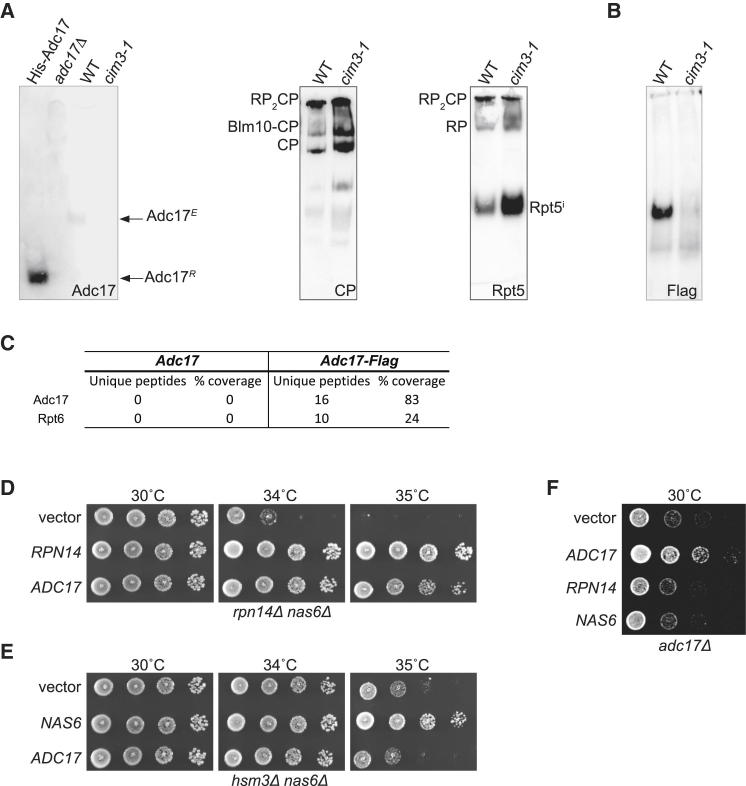
Adc17 Is Engaged in a Complex with Rpt6 Distinct from Mature Proteasome and Is Functionally Related to RP Assembly Chaperones (A) Recombinant His-Adc17 (4 ng) and yeast extracts were separated by native-PAGE (5.25%) followed by immunoblotting with Adc17 antiserum or with antibodies against CP or RP (Rpt5). Adc17^*R*^, recombinant Adc17; Adc17^*E*^, endogenous Adc17. Note that the Rpt5 species migrating in the middle of the gel are likely to be an assembly intermediate (Rpt5^i^) with increased abundance in the *cim3-1* cells. (B) Yeast extracts from Adc17-Flag cells were separated by native-PAGE (5.25%) followed by immunoblotting with Flag antibody. Note that lysates shown in (A) and (B) were run at the same time. (C) Lysates from either wild-type cells (Adc17) or Adc17-Flag cells were immunoprecipitated with anti-Flag antibodies, and immunoprecipitates were analyzed by mass spectrometry. The number of unique peptides and coverage are indicated. No Rpt6 or Adc17 peptides were identified in the Flag-immunoprecipitation on wild-type cells where Adc17 is untagged (left panel). While other proteins were detected in Flag immunoprecipitates from both Adc17 (control) and Adc17-Flag lysates, Rpt6 was the only protein that specifically coprecipitated with Adc17-Flag. (D–F) Cells were spotted in 6-fold dilution and grown at the indicated temperatures. (D) Overexpression of *ADC17* or *RPN14* suppresses the temperature-sensitive growth defect of the *rpn14Δ nas6Δ* defects mutant. (E) Overexpression of *NAS6* but not *ADC17* suppresses the temperature-sensitive growth defect of the *hsm3Δ nas6Δ* defects mutant. (F) The defects of *adc17Δ* are rescued by *ADC17* but not *RPN14* or *NAS6*. Each panel shows representative results of at least three independent experiments.

Our results described above revealed that overexpression of Adc17 rescues the proteasome defects while deletion of *ADC17* compromises proteasome integrity. However, Adc17 was not previously reported to be associated with the proteasome. To verify this, we next analyzed the mobility of Adc17 on native-PAGE followed by immunoblots. Endogenous Adc17, from wild-type cell lysates, migrated as a predominant species, which was absent from *adc17Δ* cells or *cim3-1* mutant cells and clearly distinct from mature proteasomes (Figures 4A and 4B). This confirms that Adc17 is not an integral component of proteasomes. The mobility of the endogenous Adc17 species (Adc17^*E*^) was much slower than recombinant Adc17 (Adc17^*R*^, Figure 4A). To determine whether Adc17 was engaged in a complex in cells, Adc17-Flag was immunoprecipitated and the immunoprecipitated proteins were analyzed by mass spectrometry. Rpt6 was the only protein absent from the control immunoprecipitations that specifically coprecipitated with Adc17-Flag (Figure 4C). Note that Adc17-Flag was functional (Figure S4) and that tagged protein exhibited the same mobility as the untagged Adc17 on Native-PAGE (Figure 4A). These results reveal that in wild-type cells, Adc17 is engaged in a complex with Rpt6. This complex is distinct from mature proteasome complexes and is likely to be a proteasome assembly intermediate.

### Adc17 Is Functionally Related to RP Assembly Chaperones

The results obtained so far suggested that Adc17 might play a role in RP assembly. The known RP assembly chaperones Nas2, Nas6, Rpn14, and Hsm3 have overlapping functions (Funakoshi et al., 2009, Saeki et al., 2009). We thus examined possible genetic interactions between *ADC17* and some known proteasome RP assembly chaperones, using dosage suppression as a robust experimental paradigm. The single deletion of either Rpn14, Nas2, Nas6, or Hsm3 does not cause growth defects, in contrast to combined deletions (Funakoshi et al., 2009, Le Tallec et al., 2009, Roelofs et al., 2009, Saeki et al., 2009). Among the double-deletion strains, *rpn14Δ nas6Δ* showed the most severe phenotype at elevated temperatures (Funakoshi et al., 2009, Le Tallec et al., 2009, Roelofs et al., 2009, Saeki et al., 2009), probably because both Rpn14 and Nas6 are components of the same assembly module (Rpt3-Rpt6). Having found that Adc17 forms a complex with Rpt6 and selectively suppresses the defects of the *cim3-1* mutant, we wondered whether Adc17 might be involved in the formation of the Rpt6-Rpt3 module. To assess this possibility, we examined whether Adc17 could suppress the defects of the yeast mutant lacking the two assembly chaperones of the Rpt3-Rpt6 module, Rpn14 and Nas6. Overexpression of *ADC17* suppressed the severe growth defects of *rpn14Δ nas6Δ* at 34°C and 35°C (Figure 4D). The suppression of the *rpn14Δ nas6Δ* defects by *ADC17* was robust, albeit slightly less effective than the rescue with *RPN14* (Figure 4D). This establishes that the function of Adc17 overlaps with Rpn14. Interestingly, Adc17 overexpression failed to rescue the *hsm3Δnas6Δ* defects while Nas6 did (Figure 4E). Thus, Adc17 is a selective suppressor of defects in the Nas6-Rpt3-Rpt6-Rpn14 module, whether the defects are caused by a *ts* mutation in Rpt6 or due to the lack of its assembly chaperones. In addition, we found that the growth defects of *adc17Δ* cells were rescued by *ADC17* but not *RPN14* or *NAS6* (Figure 4F). This reveals that the function of Adc17 is functionally distinct, albeit related to Rpn14. We then next examined this function in greater detail.

### Adc17 Assists Early Steps in Proteasome Biogenesis

**Figure 5:**
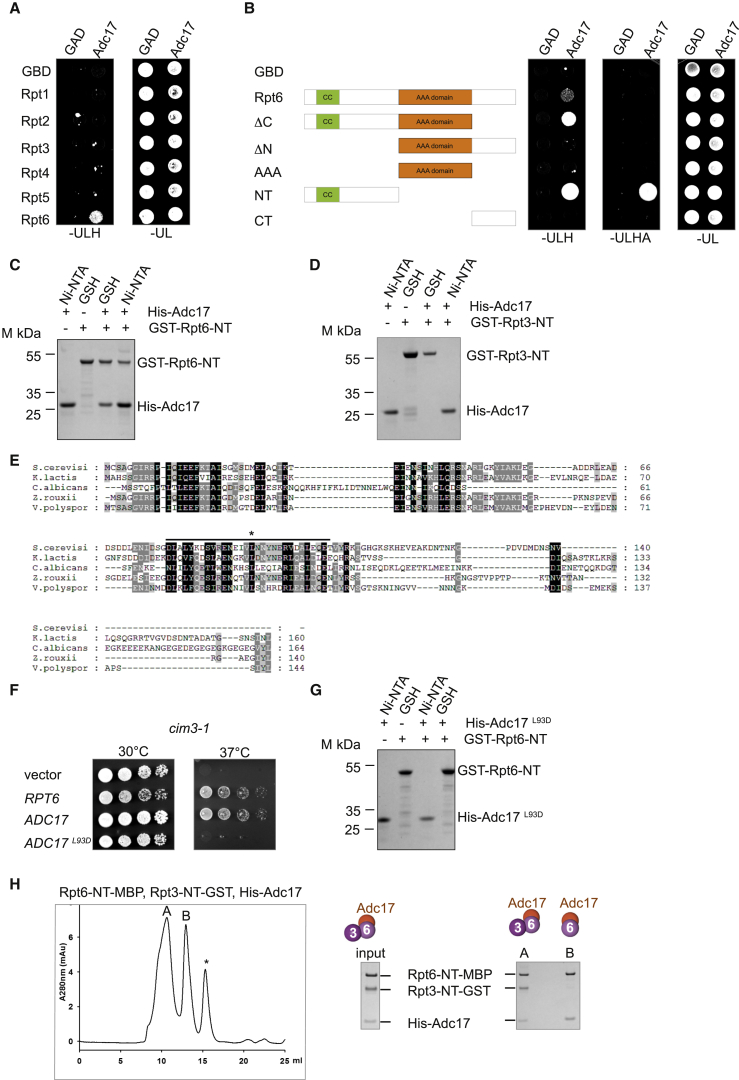
Adc17 Binds to Rpt6 and Assists Pairing of Rpt6 and Rpt3 Amino Termini (A) Yeast carrying the indicated plasmid combinations were spotted on selective medium lacking uracil, leucine, and histidine (−ULH) for yeast two-hybrid selection based on His3-auxotrophy. Growth on medium lacking uracil and leucine (−UL) served as a control. GAD, activation domain vector; GBD, DNA binding domain vector. (B) Left: schematics of Rpt6 domains. CC, coiled-coil. Right: Same as in (A) with indicated constructs. Growth on medium lacking uracil, leucine, histidine, and adenine (−ULHA) serves as stringent yeast two-hybrid selection. (C) Adc17 binds to Rpt6-NT. His-Adc17 and GST-Rpt6-NT (1–152) expressed in *E. coli* alone or together and purified on Ni-NTA (Ni) affinity resin or glutathione Sepharose 4B (GSH), resolved by SDS-PAGE, and stained with Coomassie blue. (D) Adc17 does not bind to Rpt3-NT. His-Adc17 and GST-Rpt3-NT (1–162) expressed in *E. coli* alone or together were purified on Ni-NTA affinity resin (Ni) or glutathione Sepharose 4B (GSH), resolved by SDS-PAGE, and stained with Coomassie blue. (E) Protein alignment of Adc17 from indicated species. The line indicates a predicted coiled-coil and the asterisk indicates a conserved leucine in the middle of the predicted coiled-coil. Identical or conserved residues are boxed in black and similar residues are in gray. (F) Yeast cells (*cim3-1)* transformed with the indicated plasmids or empty vector (p416 GPD) were serially diluted, spotted on selective medium, and grown at indicated temperature. (G) Same as in (C) except that a point mutant of Adc17 (L93D) was used. (H) Rpt6-NT-MBP (1–140), Rpt3-NT-GST (1–156), and His-Adc17 were coexpressed in *E. coli*, purified on amylose resin, eluted, and analyzed by gel filtration. Left: chromatogram. Right: Coomassie-stained gels showing proteins analyzed from peak fractions indicated with “A” and “B.” The identity of the proteins was confirmed by mass spectrometry. ^∗^, MBP. Representative results of at least three independent experiments are shown in each panel.

To further characterize the function of Adc17 in proteasome assembly, we next examined the interaction between Adc17 and Rpt6 in several independent experimental systems. Yeast two-hybrid analyses confirmed that Adc17 specifically interacted with Rpt6 (Figure 5A). Surprisingly, unlike all the previously known Rpt assembly factors that bind to the carboxy-terminal region of their cognate ATPase, thereby preventing binding of intermediates to the CP (Funakoshi et al., 2009, Le Tallec et al., 2009, Park et al., 2013, Roelofs et al., 2009, Saeki et al., 2009), Adc17 selectively interacted with the amino-terminal domain of Rpt6 (Rpt6-NT) (Figure 5B). When coexpressed in *E. coli*, GST-Rpt6-NT and His-Adc17 formed a complex, demonstrating that Adc17 directly interacted with Rpt6 (Figure 5C). In contrast, Adc17 did not form a complex with GST-Rpt3-NT under the same conditions (Figure 5D). Sequence analysis of Adc17 revealed the presence of a putative coiled-coil domain (Figure 5E), and analysis by circular dichroism showed that Adc17 was α-helical (Figure S5). To determine whether the coiled-coil domain in Adc17 was important for Adc17’s function, we replaced leucine 93 with an aspartate. This substitution abrogated the ability of Adc17 to rescue the *cim3-1* defects (Figure 5F) and to interact with GST-Rpt6-NT (Figure 5G), establishing the importance of this interaction for Adc17 function.

We next investigated the functional consequence of the interaction between Rpt6 and Adc17. The six ATPases, Rpt1–Rpt 6, of the RP assemble in a unique order (Förster et al., 2009, Tomko et al., 2010) upon formation of a trimer of specific pairs of ATPases (Tomko and Hochstrasser, 2013). How the highly related Rpt subunits assemble with their cognate partner in the complex cellular environment remains to be elucidated. Since Adc17 binds to the amino-terminal region of Rpt6, and since structural analyses of the proteasome have revealed that Rpt dimers are held together by the amino-terminal regions of each Rpt (Beck et al., 2012, da Fonseca et al., 2012, Lander et al., 2012), we wondered whether Adc17 played a role in the Rpt6-Rpt3 heterodimer formation. We therefore designed assays to test this possibility. Rpt3 and Rpt6 amino-terminal regions were tagged at their carboxyl termini, to keep their amino termini free, and expressed in *E. coli* in the presence or absence of Adc17. Following coexpression of Rpt6-NT-MBP and Rpt3-NT-GST from a polycistronic vector, we only recovered Rpt6-NT-MBP on amylose resins (Figure S5). Thus, under these conditions Rpt6-NT-MBP and Rpt3-NT-GST did not form a complex (Figure S5). In contrast, coexpression of Adc17 with Rpt6-NT-MBP and Rpt3-NT-GST led to the formation of two abundant complexes: A trimeric complex containing Adc17 with the Rpt6, Rpt3 derivatives and a dimeric complex containing Rpt6-NT bound to Adc17 (Figure 5H). This reveals that Adc17 is required for Rpt6 and Rpt3 amino termini to interact. Since Adc17 did not interact with Rpt3 (Figures 5A and 5D) and because in the absence of Adc17, Rpt3-NT-GST was not recovered (Figure S5), these results imply that without a productive interaction with its dedicated partner, Rpt3-NT was unstable (Figure S5). Together, these results reveal that the binding of Adc17 to Rpt6-NT renders Rpt6-NT competent to engage a productive interaction with Rpt3-NT. The assays described above establish that Adc17 assists pairing of Rpt6-Rpt3 amino termini and explains why decreased levels of Adc17 are detrimental to *cim3-1*.

### Adc17 Is Induced by Stress to Adapt Proteasome Biogenesis to the Increased Demands

Having identified the function of Adc17, the physiological importance of Adc17 remained to be elucidated. The results obtained so far revealed that the lack of Adc17 reduced cell fitness (Figure 2A), compromised proteasome integrity, and aggravated the defects of the *cim3-1* mutant (Figure 2C). Together, these results argue that Adc17 is important for cell physiology. However, a proteasome regulatory complex was recently obtained by coexpression of nine integral base subunits and the four known proteasome assembly chaperones Nas2, Nas6, Rpn14, and Hsm3 in *E. coli* (Beckwith et al., 2013). While Adc17 was dispensable in this study, our results suggest that Adc17 confers an advantage for proteasome integrity in cells. We then explored this possibility further.

**Figure 6:**
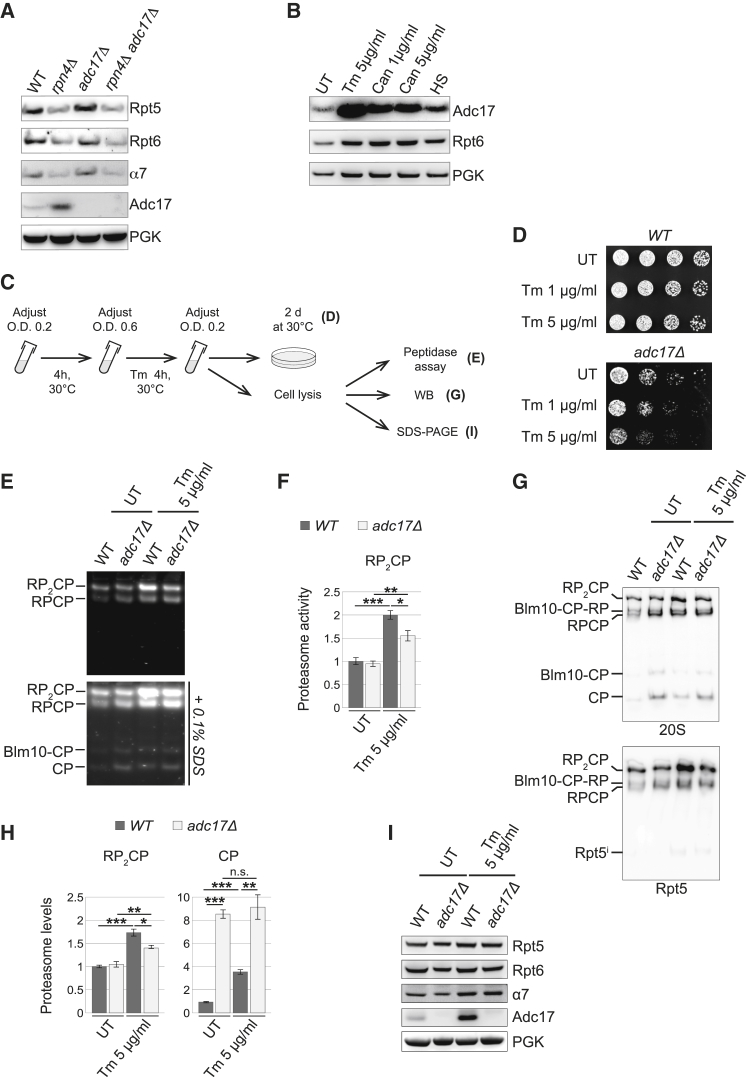
Adc17 Adapts Cells to Proteasome Stress (A) Immunoblots of total yeast extracts of cells of the indicated genotype grown at 30°C. (B) Immunoblots of total extracts of wild-type cells either left untreated (UT) or treated with tunicamycin (Tm, 5 μg/ml), treated with canavanine (Can, 1 or 5 μg/ml), or exposed to 37°C for 4 hr (HS, heat shock). Shown are results representative of an experiment repeated independently three times. (C) Cartoon depicting the experiments performed in (D), (E), (G), and (I). (D) Cells of the indicated genotype were grown in liquid cultures with or without tunicamycin at the indicated concentration, spotted in 6-fold dilutions, and grown at the indicated temperature according to the experimental workflow depicted in (C). (E) Native-PAGE (4.2%) of yeast extracts of cells of indicated genotype cultured as depicted in (C) revealed with the fluorogenic substrate Suc-LLVY-AMC. (F) Quantification of experiments such as the one shown in (E). Data are means ± SEM (n = 3). ^∗^p % 0.05, ^∗∗^p % 0.01, and ^∗∗∗^p % 0.001. (G) Immunoblots of native-PAGE (4.2%) of yeast extracts of cells of indicated genotype cultured as depicted in (C). (H) Quantification of RP_2_CP and CP from 20S immunoblots such as the one shown in (G). Data are means ± SEM (n = 3). ^∗^p % 0.05, ^∗∗^p % 0.01, and ^∗∗∗^p % 0.001. n.s., not significant. (I) Immunoblots of total extracts of cells of the indicated genotype grown as depicted in (C).

Having found that deletion of Adc17 aggravated the *cim3-1* defects (Figures 2C and 2D), we wondered whether the requirement of Adc17 might be exacerbated when the proteasome is overwhelmed. To test this possibility, we deleted *RPN4*, encoding the homeostatic transcription regulator of proteasome abundance (Xie and Varshavsky, 2001). As expected (Xie and Varshavsky, 2001), the levels of the proteasome subunits Rpt5, Rpt6, and α7 were markedly reduced in cells lacking Rpn4 (Figure 6A). In contrast, the levels of Adc17 dramatically increased in absence of Rpn4 (Figure 6A). This suggests that cells adapt to reduced levels of proteasome resulting from the deletion of *RPN4* by increasing Adc17. This result prompted us to examine whether this increase was only occurring following the deletion of RPN4 or whether the increased abundance of Adc17 represents a generic adaptive response to overwhelming demands on the proteasome. Thus, we next tested whether Adc17 levels increased upon different stresses that impose a high burden on the proteasome. Indeed, we found that treatment of yeast cells with tunicamycin, which causes accumulation of misfolded proteins in the endoplasmic reticulum, dramatically increased Adc17 levels (Figure 6B). Likewise, canavanine and heat shock also increased the abundance of Adc17. Although robust, this increase was less dramatic than that caused by tunicamycin (Figure 6B). The different stressors tested are also expected to increase proteasome levels (Xie and Varshavsky, 2001), and this was manifested here by an increase in Rpt6 levels (Figure 6B). The increased levels of Adc17 upon stress together with the increased requirement of Adc17 for the fitness of *cim3-1* (Figure 2C) suggest that the importance of Adc17 is exacerbated when the proteasome is overwhelmed.

Having found that the levels of Adc17 dramatically increased upon tunicamycin treatment, we tested whether *adc17Δ* had an increased sensitivity to tunicamycin. Note that the experimental condition used here ensured that the cells were in exponential growth phase before tunicamycin treatment (Figure 6C). We found that cells lacking Adc17 had an increased sensitivity to tunicamycin (Figure 6D). This confirms the importance of Adc17 in adapting cells to stress.

Together, our results suggest that Adc17 has evolved to facilitate proteasome assembly when the need increases, as is the case under stress conditions. To test this possibility, proteasomes were examined by in-gel peptidase assays on whole-cell lysates collected from unstressed or stressed cells, either wild-type or lacking Adc17. As expected, tunicamycin treatment increased the levels of RP_2_CP detected by in-gel peptidase assays (Figures 6E and 6F). Western blot analysis of native gels also revealed that tunicamycin increased RP_2_CP (Figures 6G and 6H). These results confirmed that the abundance of proteasomes increases upon stress. In contrast to wild-type cells, the ability of *adc17Δ* cells to increase the levels of RP_2_CP following tunicamycin treatments was compromised (Figures 6E–6H). Furthermore, *adc17Δ* cells accumulated free CP (Figures 6E–6H), revealing a defect in proteasome assembly. Importantly, the levels of the RP subunits Rpt6 or Rpt5 or the core subunit α7 were undistinguishable in wild-type and *adc17Δ* cells, both in untreated cells and following tunicamycin treatments (Figure 6I). Thus the reduced levels of RP_2_CP in *adc17Δ* cells compared to wild-type cells following tunicamycin treatment were caused by a proteasome assembly defect. Similar experiments were carried out using heat shock as a different stress inducer (Figure S6A). As seen in tunicamycin-treated cells (Figure 6E), heat shock increased the levels of 26S proteasomes detected by in-gel peptidase assays, and this increase was compromised in *adc17Δ* cells (Figures S6B–S6E). Together, these results establish that Adc17 is crucial for biogenesis of adequate proteasome levels during stress.

## Discussion

With the discovery of a stress-inducible ATPase dedicated chaperone, this study reveals how cells control homeostatic levels of proteasomes.

### Maintaining Proteasome Homeostasis

Cells have evolved numerous and often overlapping homeostatic pathways to adapt to changes in their environment and to survive under adverse conditions. The proteasome controls the degradation of a large number of cellular proteins and thereby regulates essentially all cellular processes. As cell survival depends on adequate levels of functional proteasome, cells must have evolved multiple mechanisms to adapt to the adverse conditions that compromise proteasome function or overwhelm its capacity. Such conditions include protein misfolding stress, which represents an important problem for cells and organisms, where failure to remove misfolded, aggregation-prone proteins is associated with a broad range of pathological conditions.

One adaptive response to increased demands on proteasomes is well established and relies on the transcription factor Rpn4 (Xie and Varshavsky, 2001). When the load of proteins to be degraded exceeds the capacity of the proteasome, the otherwise rapidly degraded transcription factor Rpn4 accumulates in cells, and this in turn increases expression of proteasome subunits (Xie and Varshavsky, 2001). These subunits then need to be faithfully assembled to generate a functional proteasome. While the mechanisms governing the assembly of the proteasome have been largely elucidated, how cells accomplish such a challenging task under stress conditions is unknown. Since the levels of proteasomes are known to increase under stress (Lee et al., 2004), cells must have additional mechanisms to overcome the difficult challenge that proteasome assembly represents during stress.

**Figure 7:**
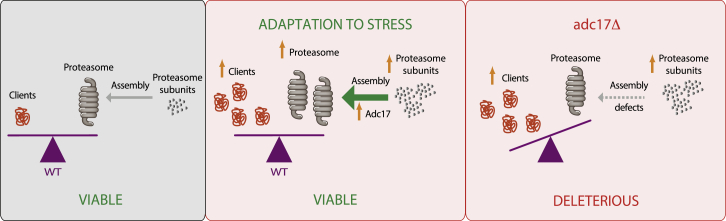
Adaptation of Proteasome Assembly to Stress Stresses (red box) increasing the abundance of client proteins and thereby overwhelming the proteasome represent a vital challenge for cells. To survive, cells adapt to stress by increasing expression of proteasome subunits. Adc17 (middle panel), a stress-inducible proteasome chaperone, promotes proteasome assembly during stress and is required to maintain vital levels of proteasomes. In *adc17Δ* cells (right panel), failure to adjust proteasome assembly to increased demands results in a deleterious imbalance between the load of protein clients and the proteolytic capacity of cells.

Through an unbiased screen in yeast, we have discovered that a previously uncharacterized protein is an effector of a stress response pathway that couples proteasome assembly to the Rpn4-mediated increased expression of its subunits. Adc17 facilitates the pairing of Rpt6 and Rpt3, an early step in proteasome biogenesis (Figures 5 and 7). Adc17 is inducible upon stress, thereby providing an opportunity to adapt proteasome assembly to the increased needs, a function that is crucial for cells to adapt to stresses (Figure 6). Importantly, unlike the proteasome subunits, Adc17’s expression is not under the control of Rpn4. Therefore, two pathways control proteasome homeostasis: one depending on Rpn4 and one depending on Adc17. While the RP assembly chaperones Nas2, Nas6, Hsm3, and Rpn14 have been studied in many different laboratories, there is no evidence that their expression might be regulated, unlike Adc17. Thus, Adc17 defines a distinct paradigm in proteasome stress response pathway.

### Adc17 and RP Assembly Chaperones: Similarities and Differences

In the recent years, there has been much progress toward understanding the assembly of the proteasome (Saeki and Tanaka, 2012, Tomko and Hochstrasser, 2013). The hexameric AAA ATPase ring of the regulatory particle (RP) forms upon assembly of three modules, each composed of a specific pair of ATPases, Rpt1-Rpt2, Rpt3-Rpt6, or Rpt5-Rpt4, bound to one or two RP assembly chaperones, Hsm3, Nas2, Nas6, and Rpn14 (Funakoshi et al., 2009, Le Tallec et al., 2009, Roelofs et al., 2009, Saeki et al., 2009). However, the mechanisms governing the formation of the three modules remain to be elucidated.

The four previously known RP assembly chaperones share a common function that is now well defined (Funakoshi et al., 2009, Le Tallec et al., 2009, Park et al., 2013, Roelofs et al., 2009, Saeki et al., 2009). Hsm3, Nas2, Nas6, and Rpn14 bind to the carboxyl termini of Rpts, thereby preventing interactions of immature complexes with the core particle (CP) (Park et al., 2013). In contrast to all known RP assembly chaperones, Nas2, Nas6, Hsm3, and Rpn14, we found that Adc17 binds to Rpt6 amino terminus. Thus, the mapping of the interaction between Rpt6 and Adc17 immediately revealed that the function of Adc17 was mechanistically distinct from the known RP assembly factors, although functionally related. Since Adc17 interaction maps to the amino terminus of Rpt6, a region predicted to be important for the selective pairing of Rpts (Beck et al., 2012), we suspected that Adc17 might be involved in the formation of the ATPase pairs. An unbiased assay with recombinant proteins revealed that productive pairing of Rpt6 and Rpt3 amino termini depends on Adc17 (Figures 5G and S4). These findings are relevant to the full-length Rpt6 protein for the following reasons. We found that Adc17 not only binds the amino terminus of Rpt6 but also forms a complex with Rpt6 full-length that we recovered from cell lysates by immunoprecipitation and confirmed this interaction in a two-hybrid assay. We also established that a point mutation in a conserved leucine in the coiled-coil region of Adc17 abolishes its ability to interact with Rpt6-NT (Figure 5G) as well as its function (Figure 5F), thereby demonstrating that the function of Adc17 is mediated by its interaction with the Rpt6 amino terminus. Interestingly, coexpression of Adc17 with Rpt6-NT and Rpt3-NT led to the formation of a heterotrimer Rpt6-Rpt3-Adc17 complex, in addition to an Rpt6-Adc17 dimer (Figure 5H), while immunoprecipitations from yeast lysates only recovered Rpt6-Adc17 complex (Figure 4C). This suggests that in cells, an Rpt6-Rpt3-Adc17 complex might be short-lived and rapidly incorporated in higher order modules. Supporting this hypothesis, Rpt5 module was detectable in wild-type cells, and the abundance of this assembly intermediate increased in the *cim3-1* mutant (Figure 3A). Thus, the formation of the Rpt3-Rpt6 module might be a rate-limiting step in proteasome assembly. Adc17, being stress inducible, provides a mechanism to finely tune the first commitment step in proteasome assembly.

Although a proteasome regulatory complex can be produced by coexpression of base subunits and the four known proteasome assembly chaperones Nas2, Nas6, Rpn14, and Hsm3 in *E. coli* (Beckwith et al., 2013), in absence of Adc17, our results demonstrate that in vivo, Adc17 confers a selective advantage for proteasome assembly. The deletion of Adc17 compromises cell fitness (Figure 2A), and this is associated with increased levels of free 20S proteasome (Figure 6H), a hallmark of proteasome assembly defects. Furthermore, the requirements of Adc17 are exacerbated under conditions of proteasome deficiencies or when proteasomes are insufficient (Figures 2C, 2D, and 6D). The recent study (Beckwith et al., 2013) suggests that it should be possible to reconstitute RP assembly with recombinant proteins and assembly factors. Such a system would be ideal to fully characterize the sequential steps in the ordered assembly of the RP and the release of proteasome chaperones from assembly intermediates, and to isolate intermediates that in cells may be too evanescent to isolate, such as the Adc17-Rpt6-Rpt3 intermediate.

Adc17 defines a paradigm of RP chaperones, which binds to the amino terminus of an Rpt to facilitate assembly with its partner (Figure S7). We have shown that Adc17 is essential to assemble the amino-terminal domains of Rpt6 and Rpt3 (Figure 5G). In the context of the full-length proteins, the function of Adc17 is also important since Adc17 is important for cell fitness and proteasome integrity but conditionally essential, as Adc17’s function partly overlaps that of Rpn14 (Figure 4D). However, under stress conditions, Adc17 becomes crucial. The interaction between Adc17 and Rpt6 seems stronger with the isolated amino-terminal domain of Rpt6 than with the full-length protein (Figure 5B), raising the possibility that this interaction might take place cotransnationally. Coincidentally, Adc17 (Tma17) was previously isolated in a systematic screen for ribosome-associated proteins (Fleischer et al., 2006).

While no sequence homologs of Adc17 have been identified in mammals so far, the conservation of proteasome assembly across species (Funakoshi et al., 2009, Kaneko et al., 2009, Le Tallec et al., 2009, Roelofs et al., 2009, Saeki et al., 2009) predicts that a functional analog ought to exist in higher eukaryotes. This is a reasonable speculation. Like Adc17, no sequence homologs were found in higher eukaryotes for the yeast HAC1, the target of the evolutionarily conserved IRE1, a key component of the unfolded protein response, while a functional homolog, XBP-1, exists (Walter and Ron, 2011). The high sequence similarities between Rpt1–Rpt6 raises the possibility that other ATPase dedicated chaperones might exist to facilitate the pairing of Rpt1 with Rpt2 and Rpt4 with Rpt5. Alternatively, the Rpt6-Rpt3 module may require a higher number of chaperones than the other modules, as the formation of this module appears to be the first and rate-limiting step in RP assembly. This study provides the basis for searching for additional regulators of proteasome assembly.

The identification of Adc17 and its functional characterization reveals that cells have mechanisms to adjust proteasome assembly to increased demands (Figure 7). Thus, Adc17 delineates an adaptive pathway to protein quality control deficiencies. As shown here, failure to adapt proteasome assembly when the demands on proteasomes increase is deleterious. Identifying pathways to survive protein quality control failure may ultimately be of therapeutic value to ameliorate the large number of pathologies associated with proteasome deficiencies.

## Experimental Procedures

### Yeast Methods and Growth Assays

All yeast manipulations were conducted according to standard protocols (Gietz and Woods, 2006). To assess growth phenotypes, exponentially growing liquid cultures expressing the indicated genes were equilibrated to an OD_600_ of 0.2, and 5 μl were spotted in serial dilutions (1/6) onto YPD or selective media as required. Plates were incubated at the indicated temperature for 2–3 days. To assess canavanine sensitivity, cells were spotted on plates lacking arginine supplemented with canavanine (1 μg/ml). Rescued cells overexpressed *ADC17* (p416 GPD), *RPN14* (p416 GPD), or wild-type RPT6 (pFL44). Yeast strains used in this study are presented in Table S1.

### Screening Procedure and Analysis

The identification of suppressors was carried out in two different screening setups. In one screening setup, *cim3-1* was transformed with the library pFL44, plated onto −Ura plates and the plates were incubated at 37°C for seven days. Clones were picked daily. For the second screening setup cells plated on −Ura plates were heat shocked at 37°C for 24 hr and then grown at 30°C for 24 hr. During the second incubation, clones were isolated. The second setup was used in two independent screens.

The plasmid of each isolated clone was extracted using a standard yeast DNA extraction procedure, and the DNA was transformed into *E. coli* DH5α (Life Technologies). Transformed *E. coli* were sent for sequencing (GATC Biotech AG). Plasmids used in this study are presented in Table S2.

### Immunoblot Analyses

Five to ten milliliters of exponentially growing cells were harvested by centrifugation at 4,000 rpm for 3 min at 4°C, washed with ice-cold water, and subsequently frozen on dry ice. Where indicated, cells were cultured in the presence of 1 μg/ml or 5 μg/ml canavanine in medium lacking arginine or in YPD supplemented with 5 μg/ml tunicamycin.

Cells were resuspended in 200 μl Rapid Protein preparation buffer (Foiani et al., 1991) (10 mM Tris-HCl [pH 7.4], 100 mM NaCl, 30 mM MgCl_2_, 1 mM DTT, two complete protease inhibitor cocktail tablets [PiC, Roche] per 50 ml, 200 μM PMSF) and transferred into clean tubes containing 200 μl acid-washed glass beads (0.5 mm; Sigma) and frozen on dry ice. Cells were lysed by vortexing twice for 5 min with intervals on ice. Subsequently, 200 μl Rapid Protein preparation buffer was added, and samples were centrifuged for 1 min at 15,000 rpm at 4°C. The supernatant was transferred to a clean tube and the remaining cell debris was removed by centrifugation for 10 min at 15,000 rpm at 4°C. Protein concentrations were measured by monitoring OD_280_, and 75 μg of total protein extract was loaded on NuPAGE 4%–12% Bis-Tris gels (Life Technologies) and resolved in MES buffer. Gel-separated protein samples were transferred to nitrocellulose membranes (Life Technologies). Membranes were incubated with antibodies to ubiquitin (1:2,000; Dako Z0458), phosphoglycerate kinase (PGK; 1:5,000) (Life Technologies), Flag (1:1,000, M2, Sigma A8592), α7 (1:1,000; Biomol PW8110), 20S (1:2,000; Biomol PW9355), Rpt5 (1:1,000; Biomol PW8245), Rpt6 (1:50; Carl Mann), and Adc17 (1:1,000; this study). Proteins were visualized by ECL Prime (GE Healthcare).

For polyubiquitin immunoblots, cells were resuspended in 200 μl lysis buffer (50 mM Tris-HCl [pH 7.4], 1 mM EDTA, 1.5 mM MgCl_2_, 1 mM DTT, PiC, 1 mM PMSF, and 50 mM N-Ethylmaleimide). Extracts from frozen pellets were prepared by heating the resuspended pellets for 10 min at 90°C. Subsequently 10 μl of 4 M acetic acid was added and samples were vortexed for 30 s. After a second incubation for 10 min at 90°C, the samples were centrifuged for 15 min at 15,000 rpm at room temperature and the supernatant was recovered.

### Native-PAGE

For native-PAGE, total yeast extracts were prepared using a freezer mill (Spex SamplePrep LLC). Exponentially growing cells were harvested, washed in ice-cold water, resuspended in lysis buffer (50 mM Tris-HCl [pH 7.4], 1 mM EDTA, 5 mM MgCl_2,_ 1 mM DTT, 2 mM ATP, and PiC), and flash-frozen in liquid nitrogen. Cells were pulverized in liquid nitrogen using the freezer mill and stored at −80°C. Yeast extracts were produced freshly by resuspending 1.5 g powder in 1.5 ml lysis buffer for 30 min at 4°C. Subsequently, the samples were centrifuged at 4,000 rpm for 10 min and the supernatants were transferred to ultracentrifuge tubes and spun in an TLA100.4 rotor (Beckman Coulter) for 27 min at 4°C at 50,000 rpm. Supernatants were recovered, avoiding the floating lipid phase and the membrane pellet. Total protein concentration of the yeast extract was measured and protein concentrations were normalized.

Native-PAGE of 4.2% or 5.25% acrylamide were prepared as described in (Elsasser et al., 2005, Suraweera et al., 2012), and 250 μg of extract was loaded for each sample.

### Immunoprecipitation and Mass Spectrometry

Wild-type and endogenously tagged Adc17-Flag were grown to exponential phase and lysed in Flag immunoprecipitation buffer (50 mM HEPES-KOH [pH 6.8], 150 mM KOAc, 5 mM MgOAc, 15% glycerol, 1 mM EDTA, 1 mM DTT, PiC, and 2 mM ATP). Yeast cell extracts were subjected to anti-Flag immunoprecipitation using anti-Flag M2 Magnetic Beads (Sigma-Aldrich), as described in Brandman et al., 2012. Immunoprecipitated proteins were eluted with 3xFlag peptides, and eluates were analyzed by mass spectrometry. The number of unique peptides and coverage of Adc17 and Rpt6 are shown.

### Yeast Two-Hybrid

Yeast two-hybrid assays were performed as described in Fleischer et al., 2006 and Saeki et al., 2009.

### Protein Expression and Purification in *E. coli*

Constructs were transformed into BL21-Gold competent cells and selected on ampicillin- and/or kanamycin-containing plates (100 mg/l and 50 mg/l, respectively). Bacteria were grown in Luria-Bertani (LB) medium containing the appropriate antibiotics at 37°C up to an OD_600_ of approximately 0.6. Expression was induced by addition of 0.1 mM IPTG for 3 hr at 30°C. Cultures were harvested and cell pellets were resuspended in lysis buffer (25 mM Tris-HCl [pH 8.0], 300 mM NaCl, 10% glycerol, PiC, and 0.2 mM PMSF), frozen in liquid nitrogen, thawed slowly on ice, and lysed by sonication. Bacterial lysates were centrifuged at 15,000 *g* at 4°C for 20 min and the supernatant was collected. The proteins and protein complexes were purified either on Ni-NTA Sepharose (QIAGEN) or Glutathione Sepharose 4B (GE Healthcare) and analyzed on NuPAGE SDS-PAGE 4%–12% Bis-Tris gels (Life Technologies) stained with Coomassie blue. The Ni-NTA resin was equilibrated in lysis buffer plus 20 mM imidazole and proteins were eluted with 25 mM Tris-HCl (pH 8.0), 100 mM NaCl, 10% glycerol, 250 mM imidazlole. The glutathione resin was equilibrated in lysis buffer and proteins were eluted with 25 mM Tris-HCl (pH 8.0), 100 mM NaCl, 10% glycerol, 5 mM glutathione.

Rpt6-NT-MBP and Rpt3-NT-GST were coexpressed in *E. coli* with or without His6x-Adc17 using the pOPC/pOPT polycistronic plasmids (Förster et al., 2009, Ghislain et al., 1993, Tan, 2001, Tomko et al., 2010) and purified on amylose resin (New England Biolabs). Expression was performed as described above. The protein complexes were purified on amylose resin (New England Biolabs) equilibrated in lysis buffer and eluted with 25 mM Tris-HCl (pH 8.0), 100 mM NaCl, 10% glycerol, 20 mM maltose, followed by gel filtration analysis using a 24 ml Superdex 200 10/300 GL column (GE Healthcare). One hundred microliters of each complex was loaded onto the column at a flow rate of 0.4 ml/min, and 0.3 ml fractions were eluted in column buffer (25 mM Tris-HCl [pH 8.0], 100 mM NaCl). An equal volume (10 μl) from each fraction was loaded onto NuPAGE SDS-PAGE 4%–12% Bis-Tris gels (Life Technologies).

### Antibody Production

For the production of anti-Adc17 antibody, His-Adc17 was expressed in *E. coli*, purified using Ni-NTA affinity chromatography. The His-tag was cleaved and the protein was purified by gel filtration. Purified proteins were injected to two rabbits, and antisera were used at dilutions of 1:1,000.

### Circular Dichroism

Spectra (190–260 nm) of 18 μg of Adc17 protein in 5 mM Tris-HCl (pH 8.0), 50mM NaCl were recorded on a JASCO J-810 spectropolarimeter.

### RNA Isolation and Gene Expression Analysis

Yeast cells were harvested during exponential growth phase and cell wall was digested with Zymolyase (Zymo research). RNA was extracted by high pure RNA isolation kit (Roche) according to the manufacturer’s instructions. RNA quality and quantity were assessed by NanoDrop 2000c spectrophotometer (Thermo Fisher Scientific), and 500 ng of mRNA was reverse-transcribed to cDNA using an iScript cDNA synthesis kit (Bio-Rad). Quantitative PCR was performed with primers (*RPT6* forward, TTCCATTGGCTCTACTCGTG; *RPT6* reverse, AAACCCGTCCAATTGGTTTA; *TAF10* forward, ATATTCCAGGATCAGGTCTTCCGTAGC; *TAF10* reverse, GTAGTCTTCTCATTCTGTTGATGTTGTTGTTG) using SYBR Select Master Mix (Applied Biosystems) on a Rotor-Gene 6000 and analyzed using Corbett Rotor Gene Software (Corbett Life Science). Expression of *RPT6* was normalized to housekeeping gene *TAF10*, and fold change was measured using the Pfaffl equation and expressed relative to wild-type control. Data shown are averages of biological triplicates from each strain.

## Author Contributions

A.H. performed the screen, discovered Adc17, and characterized the *cim3-1* strain and Adc17 in yeast. Z.Z. elucidated the function of Adc17. A.R. established the importance of Adc17 for cell fitness.

## Supplemental Information

Document S1. Figures S1–S7 and Tables S1 and S2

Document S2. Article plus Supplemental Information

## References

[bib1] Beck F., Unverdorben P., Bohn S., Schweitzer A., Pfeifer G., Sakata E., Nickell S., Plitzko J.M., Villa E., Baumeister W., Förster F. (2012). Near-atomic resolution structural model of the yeast 26S proteasome. Proc. Natl. Acad. Sci. USA.

[bib2] Beckwith R., Estrin E., Worden E.J., Martin A. (2013). Reconstitution of the 26S proteasome reveals functional asymmetries in its AAA+ unfoldase. Nat. Struct. Mol. Biol..

[bib3] Brandman O., Stewart-Ornstein J., Wong D., Larson A., Williams C.C., Li G.-W., Zhou S., King D., Shen P.S., Weibezahn J. (2012). A ribosome-bound quality control complex triggers degradation of nascent peptides and signals translation stress. Cell.

[bib4] Cuanalo-Contreras K., Mukherjee A., Soto C. (2013). Role of protein misfolding and proteostasis deficiency in protein misfolding diseases and aging. Int. J. Cell Biol..

[bib5] da Fonseca P.C., He J., Morris E.P. (2012). Molecular model of the human 26S proteasome. Mol. Cell.

[bib6] Elsasser S., Schmidt M., Finley D.D. (2005). Characterization of the proteasome using native gel electrophoresis. Methods Enzymol..

[bib7] Finley D.D. (2009). Recognition and processing of ubiquitin-protein conjugates by the proteasome. Annu. Rev. Biochem..

[bib8] Fleischer T.C., Weaver C.M., McAfee K.J., Jennings J.L., Link A.J. (2006). Systematic identification and functional screens of uncharacterized proteins associated with eukaryotic ribosomal complexes. Genes Dev..

[bib9] Foiani M., Cigan A.M., Paddon C.J., Harashima S., Hinnebusch A.G. (1991). GCD2, a translational repressor of the GCN4 gene, has a general function in the initiation of protein synthesis in Saccharomyces cerevisiae. Mol. Cell. Biol..

[bib10] Förster F., Lasker K., Beck F., Nickell S., Sali A., Baumeister W. (2009). An atomic model AAA-ATPase/20S core particle sub-complex of the 26S proteasome. Biochem. Biophys. Res. Commun..

[bib11] Funakoshi M., Tomko R.J., Kobayashi H., Hochstrasser M. (2009). Multiple assembly chaperones govern biogenesis of the proteasome regulatory particle base. Cell.

[bib12] Ghislain M., Udvardy A., Mann C. (1993). S. cerevisiae 26S protease mutants arrest cell division in G2/metaphase. Nature.

[bib13] Gietz R.D., Woods R.A. (2006). Yeast transformation by the LiAc/SS Carrier DNA/PEG method. Methods Mol. Biol..

[bib14] Goldberg A.L. (2007). Functions of the proteasome: from protein degradation and immune surveillance to cancer therapy. Biochem. Soc. Trans..

[bib15] Hanna J., Finley D.D. (2007). A proteasome for all occasions. FEBS Lett..

[bib16] Heinemeyer W., Kleinschmidt J.A., Saidowsky J., Escher C., Wolf D.H. (1991). Proteinase yscE, the yeast proteasome/multicatalytic-multifunctional proteinase: mutants unravel its function in stress induced proteolysis and uncover its necessity for cell survival. EMBO J..

[bib17] Hershko A., Ciechanover A. (1998). The ubiquitin system. Annu. Rev. Biochem..

[bib18] Kaneko T., Hamazaki J., Iemura S., Sasaki K., Furuyama K., Natsume T., Tanaka K., Murata S. (2009). Assembly pathway of the Mammalian proteasome base subcomplex is mediated by multiple specific chaperones. Cell.

[bib19] Kim Y.E., Hipp M.S., Bracher A., Hayer-Hartl M., Hartl F.U. (2013). Molecular chaperone functions in protein folding and proteostasis. Annu. Rev. Biochem..

[bib20] Kisselev A.F., Goldberg A.L. (2001). Proteasome inhibitors: from research tools to drug candidates. Chem. Biol..

[bib21] Kisselev A.F., Goldberg A.L. (2005). Monitoring activity and inhibition of 26S proteasomes with fluorogenic peptide substrates. Methods Enzymol..

[bib22] Lander G.C., Estrin E., Matyskiela M.E., Bashore C., Nogales E., Martin A. (2012). Complete subunit architecture of the proteasome regulatory particle. Nature.

[bib23] Le Tallec B., Barrault M.B., Guérois R., Carré T., Peyroche A. (2009). Hsm3/S5b participates in the assembly pathway of the 19S regulatory particle of the proteasome. Mol. Cell.

[bib24] Lee C.-S., Tee L.Y., Warmke T., Vinjamoori A., Cai A., Fagan A.M., Snider B.J. (2004). A proteasomal stress response: pre-treatment with proteasome inhibitors increases proteasome activity and reduces neuronal vulnerability to oxidative injury. J. Neurochem..

[bib25] Morimoto R.I. (2011). The heat shock response: systems biology of proteotoxic stress in aging and disease. Cold Spring Harb. Symp. Quant. Biol..

[bib26] Murata S., Yashiroda H., Tanaka K. (2009). Molecular mechanisms of proteasome assembly. Nat. Rev. Mol. Cell Biol..

[bib27] Navon A., Ciechanover A. (2009). The 26 S proteasome: from basic mechanisms to drug targeting. J. Biol. Chem..

[bib28] Park S., Li X., Kim H.M., Singh C.R., Tian G., Hoyt M.A., Lovell S., Battaile K.P., Zolkiewski M., Coffino P. (2013). Reconfiguration of the proteasome during chaperone-mediated assembly. Nature.

[bib29] Roelofs J., Park S., Haas W., Tian G., McAllister F.E., Huo Y., Lee B.H., Zhang F., Shi Y., Gygi S.P., Finley D. (2009). Chaperone-mediated pathway of proteasome regulatory particle assembly. Nature.

[bib30] Rubin D.M., Glickman M.H., Larsen C.N., Dhruvakumar S., Finley D.D. (1998). Active site mutants in the six regulatory particle ATPases reveal multiple roles for ATP in the proteasome. EMBO J..

[bib31] Saeki Y., Tanaka K. (2012). Assembly and function of the proteasome. Methods Mol. Biol..

[bib32] Saeki Y., Toh-E A., Kudo T., Kawamura H., Tanaka K. (2009). Multiple proteasome-interacting proteins assist the assembly of the yeast 19S regulatory particle. Cell.

[bib33] Schwartz A.L., Ciechanover A. (2009). Targeting proteins for destruction by the ubiquitin system: implications for human pathobiology. Annu. Rev. Pharmacol. Toxicol..

[bib34] Sherman M.Y., Goldberg A.L. (2001). Cellular defenses against unfolded proteins: a cell biologist thinks about neurodegenerative diseases. Neuron.

[bib35] Soto C. (2003). Unfolding the role of protein misfolding in neurodegenerative diseases. Nat. Rev. Neurosci..

[bib36] Suraweera A., Münch C., Hanssum A., Bertolotti A. (2012). Failure of amino acid homeostasis causes cell death following proteasome inhibition. Mol. Cell.

[bib37] Tan S. (2001). A modular polycistronic expression system for overexpressing protein complexes in Escherichia coli. Protein Expr. Purif..

[bib38] Tanaka K., Mizushima T., Saeki Y. (2012). The proteasome: molecular machinery and pathophysiological roles. Biol. Chem..

[bib39] Tomko R.J.J., Hochstrasser M. (2013). Molecular architecture and assembly of the eukaryotic proteasome. Annu. Rev. Biochem..

[bib40] Tomko R.J., Funakoshi M., Schneider K., Wang J., Hochstrasser M. (2010). Heterohexameric ring arrangement of the eukaryotic proteasomal ATPases: implications for proteasome structure and assembly. Mol. Cell.

[bib41] Walter P., Ron D. (2011). The unfolded protein response: from stress pathway to homeostatic regulation. Science.

[bib42] Xie Y., Varshavsky A. (2001). RPN4 is a ligand, substrate, and transcriptional regulator of the 26S proteasome: a negative feedback circuit. Proc. Natl. Acad. Sci. USA.

