# Supplementary material for: An Inducible Chaperone Adapts Proteasome Assembly to Stress
Source: Mol Cell. 2014 Aug 21;55(4):566–77. doi: 10.1016/j.molcel.2014.06.017 (PMC4148588; doi:10.1016/j.molcel.2014.06.017)
Supplement: Document S1. Figures S1–S7 and Tables S1 and S2 [file mmc1.pdf]

Molecular Cell, Volume *55*

Supplemental Information

An Inducible Chaperone Adapts Proteasome Assembly to Stress

Ariane Hanssum, Zhen Zhong, Adrien Rousseau, Agnieszka Krzyzosiak, Anna Sigurdardottir, and Anne Bertolotti

Supplemental figures and legends

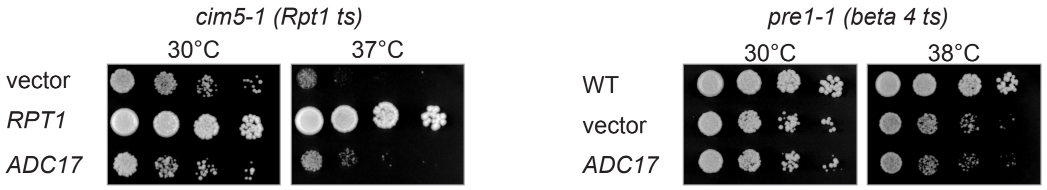

Figure S1, Related to Figure 1. Adc17 does not suppress the growth defects of the proteasome mutants *cim5-1* and *pre1-1*

Assessment of growth of *cim5-1* or *pre1-1* cells transformed with the indicated plasmids or empty vector. Cells were spotted in sixfold dilution and grown at the indicated temperatures.

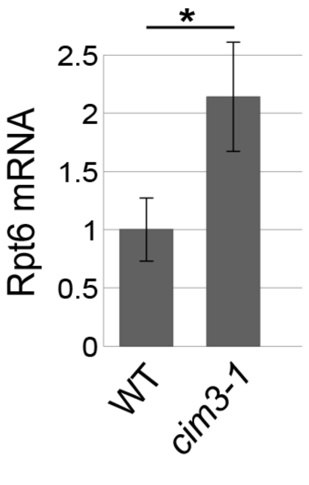

Figure S2, Related to Figure 2. Relative abundance of Rpt6 mRNA in WT and *cim3-1* cells

The abundance of Rpt6 mRNA were measured by qRT-PCR in wild-type (WT) and *cim3-1* cells. Expression of *RPT6* was normalized to *TAF10*. Data are means ± s.e.m. (n = 3).

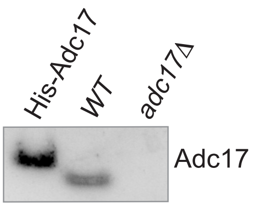

Figure S3, Related to Figure 3. Characterization of Adc17 antibody

Immunoblot of recombinant His-Adc17 (2 ng) or protein lysates (25 µg) of wild-type (WT) or *adc17Δ* cells, revealed by Adc17 antiserum.

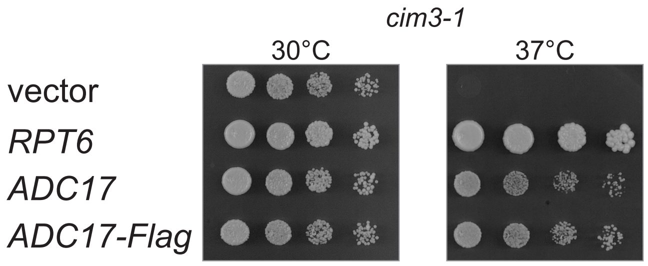

Figure S4, Related to Figure 4. Adc17-Flag is functional.

Adc17-Flag is functional. Growth of *cim3-1* cells transformed by the indicated plasmids or empty vector. Cells were spotted in sixfold dilution and grown at 30 or 37ºC.

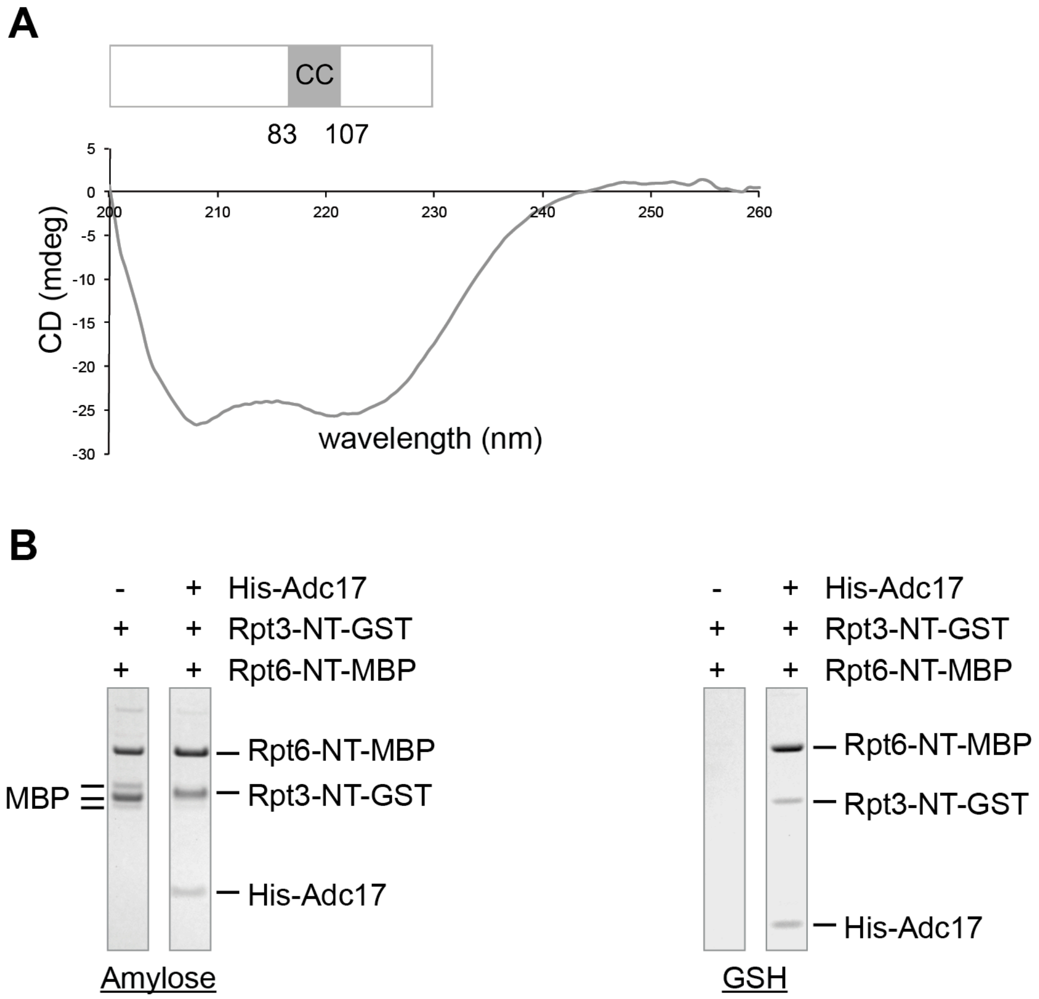

Figure S5, Related to Figure 5. The formation of a complex between Rpt6-NT and Rpt3-NT requires Adc17

A) Top: Schematic of Adc17. Bottom: Alpha-helical structure of Adc17 revealed by circular dichroism (CD).

(B) The formation of a complex between Rpt6-NT-GST and Rpt3-NT-GST depends on Adc17. Rpt6-NT–MBP (1-140) and Rpt3-NT-GST (1—156) were co-expressed in *E. coli* in the presence of His-Adc17 where indicated. Lysates were purified on amylose or glutathione sepharose resin before elution and gel analysis. Note that in absence of Adc17, Rpt3-NT-GST was unstable. Gels were stained with Coomassie Blue. The identity of the proteins was confirmed by mass spectrometry.

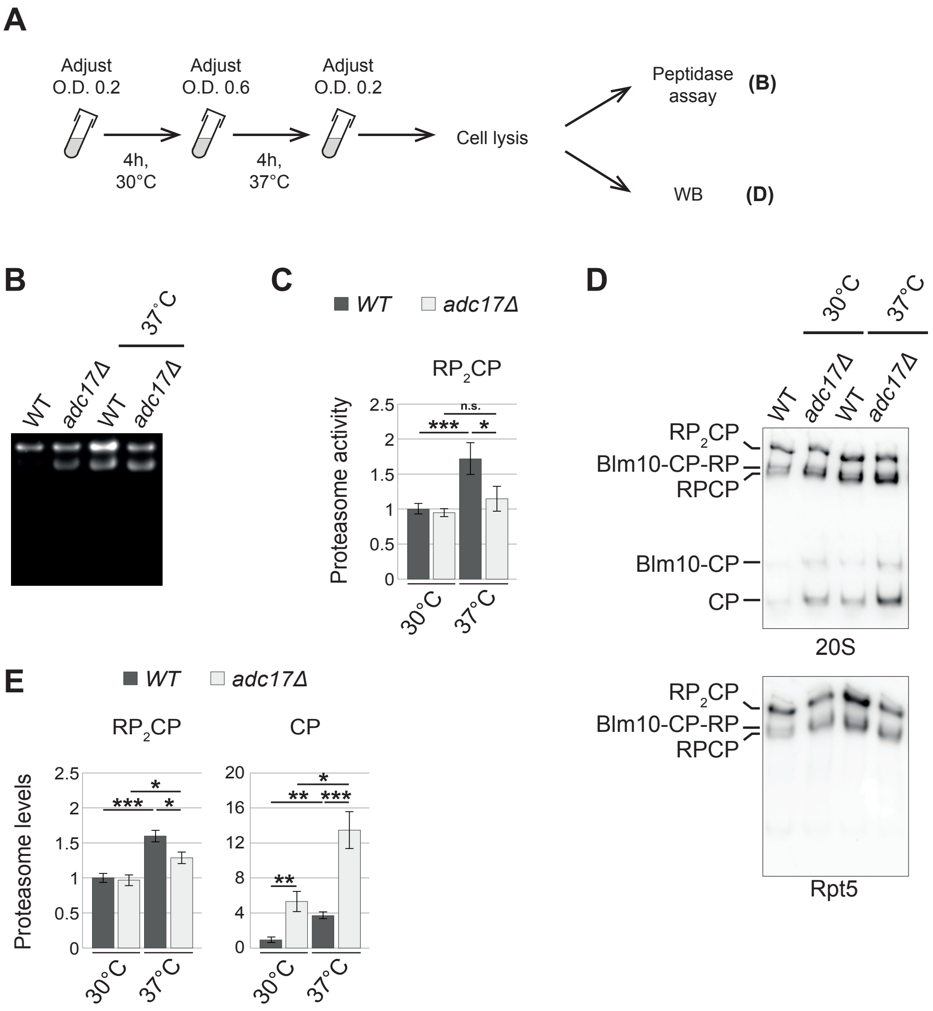

Figure S6, Related to Figure 6. Adc17 adapts proteasome assembly to heat stress

(A) Cartoon depicting the experiments performed in (B-E).

(B) Native-PAGE (4.2%) of yeast extracts of cells of indicated genotype cultured as depicted in (A) revealed with the fluorogenic substrate Suc-LLVY-AMC.

(C) Quantification of experiments such as the one shown in (B). Data are means ± s.e.m. (n = 3). *p % 0.05 and ***p % 0.001. n.s.: not significant.

(D) Immunoblots of native-PAGE (4.2%) of yeast extracts of cells of indicated genotype cultured as depicted in (A).

(E) Quantification of RP_2CP and CP from 20S immunoblots such as the one shown in (D). Data are means ± s.e.m. (n = 3). *p % 0.05, **p % 0.01, and ***p % 0.001.

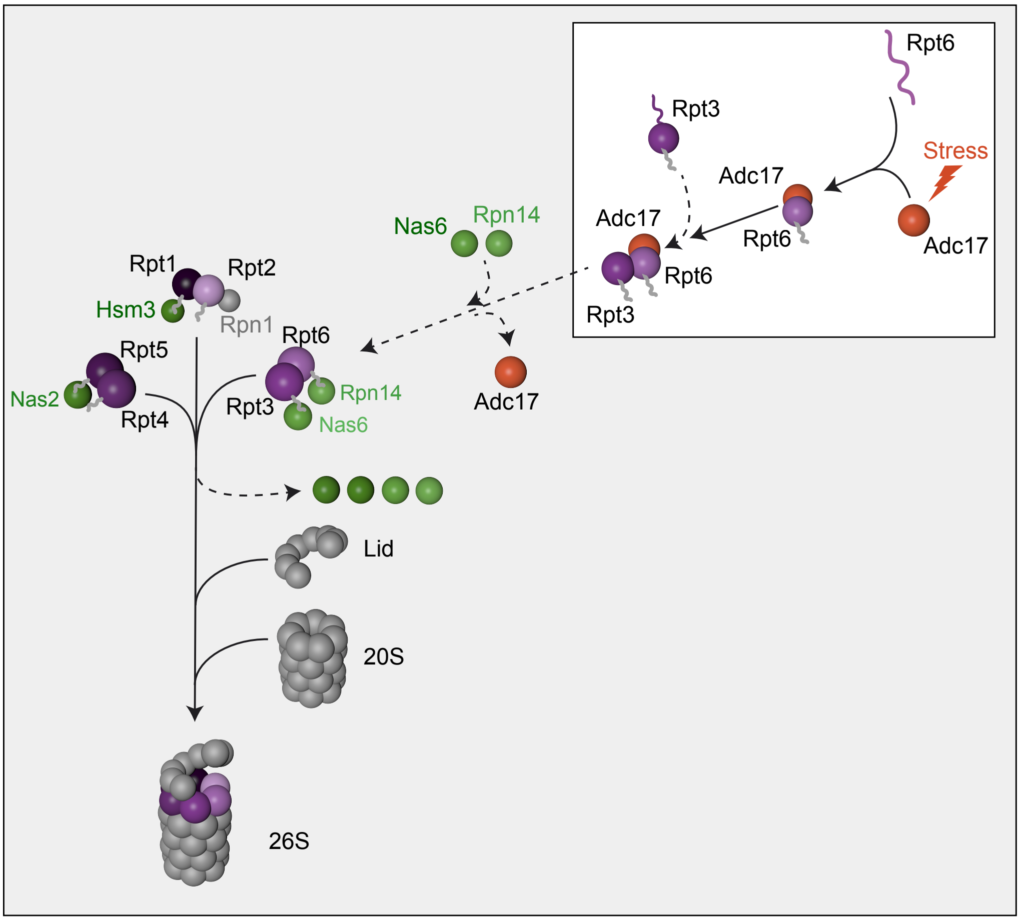

Figure S7, Related to Figure 7. Model depicting the function of Adc17 in adapting RP assembly to stress.

Adc17, a stress-induced proteasome-assembly factor, assists the productive pairing of Rpt6-Rpt3 amino-termini. Adc17 selectively binds to Rpt6 amino-terminus thereby enabling Rpt6 to engage a productive interaction with Rpt3 and initiate proteasome RP assembly. Adc17's function is crucial for cell survival during stress. The findings reported in this manuscript (white box) are presented in the context of the regulatory particle assembly pathway (see text for references).

Table S1 Yeast strains used in this study.

Name	Genotype	Source and reference
WT (S288C)	MAT**a** *ura3-52 trp14Δ63 his3Δ200 leu2Δ1 lys2-801 ade2-101*	Ghislain *et al*., 1993
cim3-1 (rpt6)	Phil Hieter YPH background, CMY 819 YPH499 (S288C) MAT**a** *cim3-1 ura3-52 trp14Δ63 his3Δ200 leu2Δ1 lys2-8*	Ghislain *et al*., 1993
cim5-1 (rpt1)	Phil Hieter YPH background, CMY 819 YPH499 (S288C) MAT**a** *cim5-1 ura3-52 trp14Δ63 his3Δ200 leu2Δ1 lys2-8*	Ghislain *et al*., 1993
WT (WCG4a)	MAT**a** *ura3 leu2-3,112 his3-11,15 rad5-535 CanS GAL2*	Heinemeyer *et al.*, 1991
pre1-1	MAT**a** *pre1-1 ura3 leu2-3,112 his3-11,15 rad5-535 CanS GAL2*	Heinemeyer *et al.*, 1991
adc17Δ (S288C)	MAT**a** *ura3-52 trp14Δ63 his3Δ200 leu2Δ1 lys2-801 ade2-101 adc17::kanMx*	This study
adc17Δ cim3-1	MAT**a** *cim3-1 ura3-52 trp14Δ63 his3Δ200 leu2Δ1 lys2-801 ade2-101 adc17::kanMx*	This study
adc17-FLAG (*WT* S288C)	MAT**a** *ura3-52 trp14Δ63 his3Δ200 leu2Δ1 lys2-801 ade2-101 adc17::adc17-FLAG3x-loxP*	This study
adc17-FLAG cim3-1	MAT**a** *cim3-1 ura3-52 trp14Δ63 his3Δ200 leu2Δ1 lys2-801 ade2-101 adc17::adc17-FLAG3x-loxP*	This study
PJ69-4A	MAT**a** *trp1-901 leu2-3,113 ura3-52 his3-200 Δgal4 Δgal80 LYS2::GAL1his3 GAL2-ADE2*	James *et al.*, 1996
rpn14Δ nas6Δ (BY4741)	MAT**a** his3Δ1 leu2Δ0 met15Δ0 ura3Δ0 rpn14::kanMx nas6::URA3	This study
hsm3Δ nas6Δ (BY4741)	MAT**a** his3Δ1 leu2Δ0 met15Δ0 ura3Δ0 hsm3::kanMx nas6::URA3	This study
BY4741	MAT**a** *his3Δ1 leu2Δ0 met15Δ0 ura3Δ0*	Thermo Scientific Open Biosyste
adc17Δ (BY4741)	MAT**a** *his3Δ1 leu2Δ0 met15Δ0 ura3Δ0 adc17::kanMx*	Thermo Scientific Open Biosyste
rpn4Δ (BY4741)	MAT**a** *his3Δ1 leu2Δ0 met15Δ0 ura3Δ0 rpn4::kanMx*	Thermo Scientific Open Biosyste
rpn4Δ adc17Δ (BY4741)	MAT**a** *his3Δ1 leu2Δ0 met15Δ0 ura3Δ0 rpn4::kanMx adc17::his5*	This study

Table S2 Plasmids used in this study

Name	Insert	Vector	Source and reference
AV54	His6x-ADC17	pET28a(+)pp	This study
AV57	GST-RPT3_NT (1-162)	pGEX4T3	This study
AV62	His6x-ADC17_L93D	pET28a(+)pp	This study
AV64	GST-RPT6_NT (1-152)	pGEX4T3	This study
P3	RPT3_NT(1-156)_GST + RPT6_NT(1-140)_MBP	pOPC	This study
AVY62	ADC17	pRS426	This study
AVY80	ADC17	p416 GPD	This study
AVY94	NAS6	p415 GPD	This study
AVY97	ADC17	p415 GPD	This study
AVY98	RPN14	p415 GPD	This study
AVY105	ADC17-FLAG3x	p416 GPD	This study
AVY119	ADC17	pGAD-C1	This study
AVY123	ADC17_L93D	p416 GPD	This study
AVY131	ADC17_L93D	pGADC1	This study
KONAp114	NAS2	pGAD-C1	Saeki et al., 2009
KONAp115	NAS6	pGAD-C1	Saeki et al., 2009
KONAp116	RPN14	pGAD-C1	Saeki et al., 2009
KONAp117	HSM3	pGAD-C1	Saeki et al., 2009
KONAp118	RPT1	pGBDU-C1	Saeki et al., 2009
KONAp124	RPT2	pGBDU-C1	Saeki et al., 2009
KONAp125	RPT3	pGBDU-C1	Saeki et al., 2009
KONAp131	RPT4	pGBDU-C1	Saeki et al., 2009
KONAp132	RPT5	pGBDU-C1	Saeki et al., 2009
KONAp138	RPT6	pGBDU-C1	Saeki et al., 2009
KONAp139	RPT6ΔC (1-319)	pGBDU-C1	Saeki et al., 2009
KONAp140	RPT6ΔN (141-405)	pGBDU-C1	Saeki et al., 2009
KONAp141	RPT6AAA (141-319)	pGBDU-C1	Saeki et al., 2009
KONAp142	RPT6NT (1-140)	pGBDU-C1	Saeki et al., 2009
KONAp143	RPT6CT (320-405)	pGBDU-C1	Saeki et al., 2009
